# Corneal topographic changes following retinal surgery

**DOI:** 10.1186/1471-2415-4-10

**Published:** 2004-08-03

**Authors:** Rajesh Sinha, Namrata Sharma, Lalit Verma, RM Pandey, Rasik B Vajpayee

**Affiliations:** 1 Rajendra Prasad Centre for Ophthalmic Sciences, All India Institute of Medical Sciences, New Delhi, India; 2 Department of Biostatistics, All India Institute of Medical Sciences, New Delhi, India

**Keywords:** anterior corneal elevation, posterior corneal elevation, scleral buckling, retinal surgery

## Abstract

**Background:**

To study the effect of retinal/ vitreoretinal surgeries on corneal elevations.

**Methods:**

Patients who underwent retinal/ vitreoretinal surgeries were divided into 3 groups. Scleral buckling was performed in 11 eyes (Group 1). In 8 (25%) eyes, vitreoretinal surgery was performed along with scleral buckling (Group 2). In 12 eyes, pars plana vitrectomy was performed for vitreous hemorrhage (Group 3). An encircling element was used in all the eyes. The parameters evaluated were best-corrected visual acuity (BCVA), change in axial length, and corneal topographic changes on Orbscan topography system II, preoperative and at 12 weeks following surgery.

**Results:**

There was a statistically significant increase in anterior corneal elevation in all the three groups after surgery (p = 0.003, p = 0.008 & p = 0.003 respectively). The increase in posterior corneal elevation was highly significant in all the three groups after surgery (p = 0.0000, p = 0.0001 & p = 0.0001 respectively). The increase in the posterior corneal elevation was more than the increase in the anterior elevation and was significant statistically in all the three groups (group I: p = 0.02; group II: p = 0.01; group III: p = 0.008).

**Conclusions:**

Retinal/ vitreoretinal surgeries cause a significant increase in the corneal elevations and have a greater effect on the posterior corneal surface.

## Background

Changes in corneal curvature and axial length have been reported following scleral buckling procedure using keratometer [1-7]. Videokeratography has also been used to assess the corneal surface changes after buckling procedure[8].

All these studies have reported a change in the corneal curvature in its anterior surface. However, no study has been performed to evaluate the posterior corneal topographic changes with scanning slit topography system following retinal surgeries. Hence, we made an attempt to study the effect of various retinal surgeries on posterior corneal surface using Orbscan II topography system.

## Methods

A prospective study was performed by enrolling patients admitted in the Retina Services of Rajendra Prasad Centre for Ophthalmic Sciences, New Delhi for retinal/ vitreoretinal surgeries. Thirty one eyes of 31 patients who underwent retinal/ vitreoretinal surgery and came for regular follow-up as per schedule during the period between December 2001 and October 2002 were included in the study. Only those patients were included who had not undergone any previous ocular surgery & did not have any corneal pathology. An informed consent was obtained from all the patients.

The patients were evaluated preoperatively on parameters of best-corrected visual acuity (BCVA), axial length (AL) measured by Ultrasound A-scan instrument (Sonomed, Inc., NY) and detailed corneal examination on slit-lamp biomicroscope and on Orbscan topography system II (Bausch and Lomb, Salt Lake City, Utah). The parameters that were evaluated by Orbscan topography system were anterior elevation, posterior elevation and simulated keratometry.

All surgeries were performed by a single surgeon (LV) under local anesthesia by peribulbar injection of 6 ml of 2% xylocaine and 2 ml of 0.5% bupivacaine. Eyes with fresh retinal detachment with clear media and absence of advanced proliferative vitreoretinopathy underwent scleral buckling procedure (Group I, n = 11). In all these eyes, the break/ s were localized, cryotherapy was performed and subretinal fluid was drained. Only circumferential buckle of silicone of style 276 (Labtician Ophthalmics, Inc., Oakville, Canada) was used and radial buckle or sponge was not used in any eye. The size of the buckle was 90° to 360° depending upon the requirement in individual cases. Encircling element of style 240 (Labtician Ophthalmics, Inc., Oakville, Canada) was used in all the eyes undergoing scleral buckling.

In eyes with associated vitreous hemorrhage or advanced proliferative vitreoretinopathy changes along with retinal detachment, vitreoretinal surgery was performed along with scleral buckling (Group II, n = 8). Pars plana vitrectomy was performed and vitreoretinal membranes were removed either by peeling or by segmentation or delamination. Air fluid exchange was performed followed by Air-Silicone oil exchange. In eyes with only vitreous hemorrhage without the presence of retinal detachment, only pars plana vitrectomy was performed (Group III, n = 12). An encircling element of style 240 (Labtician Ophthalmics, Inc., Oakville, Canada) was used in all these eyes to counter the anterior traction that could not be fully released by vitrectomy in order to avoid lens damage.

The intraoperative details including the nature of surgery, size of the buckle, encircling element, drainage of subretinal fluid, vitrectomy & use of silicone oil or gas, were noted. Postoperative treatment included topical ciprofloxacin 0.3% QID, topical dexamethasone 0.1% QID and topical Homatropine 2% QID.

The patients were evaluated at 12 weeks following surgery on similar (preoperative) parameters.

### Statistical analysis

The data of all the patients were managed on an excel spreadsheet. All the entries were checked for any possible keyboard error. Preoperative and postoperative measurements in the three retinal surgery groups were summarized by mean and standard deviation. Changes following surgery within each group were assessed using paired 't' test. Preoperative and postoperative values in the three groups were compared using one way analysis of variance (ANOVA), followed by bon ferroni correction for multiple comparison. For the three retinal surgery groups, median was computed for increase in various parameters due to surgery. Kruskal Wallis one way analysis of variance was used to compare median increase in the three groups. STATA 7.0 statistical software was used for data analysis. In this study, p-value smaller than 0.05 was considered as statistically significant.

## Results

The mean age of the patients was 45.96 ± 15.17 (range: 18–78) years and majority (83.87%) of the patients (N = 31) were males. Of these, right eye was operated in 19 (61.29%) patients.

The mean preoperative best-corrected visual acuity (BCVA) was hand motion close to face in 26 eyes; counting finger near to face in 4 eyes and 1/60 on snellen's acuity chart in 1 eye. The mean decimal postoperative BCVA was 0.20 ± 0.12 at 12 weeks follow-up after surgery.

The mean preoperative anterior corneal elevation as recorded by Orbscan II topography system in group I was 0.006 ± 0.007 mm, which increased to 0.024 ± 0.013 mm at 12 weeks after surgery (p= 0.003). In group II, it increased from 0.009 ± 0.006 mm preoperatively to 0.021 ± 0.010 mm at 12 weeks (p= 0.008) and in group III, it increased from 0.003 ± 0.004 mm preoperatively to 0.012 ± 0.007 mm at 12 weeks follow-up (p = 0.003). On comparative evaluation between the groups, the change in anterior corneal elevation was significant between group I and III (p = 0.04).

The mean posterior elevation in group I increased from a preoperative value of 0.016 ± 0.010 mm to 0.043 ± 0.007 mm at 12 weeks after surgery (p= 0.0000). In group II, it increased from 0.014 ± 0.006 mm preoperatively to 0.043 ± 0.007 mm at 12 weeks (p= 0.0001) and in group III it increased from a preoperative value of 0.012 ± 0.005 mm to 0.029 ± 0.006 mm at 12 weeks after surgery (p = 0.0001). A comparative analysis between the groups indicated that the increase in posterior corneal elevation between groups I & III and groups II & III were found to be highly significant (I vs III: p= 0.001; II vs III: p= 0.001).

Again, the increase in the posterior corneal elevation was greater than the increase in the anterior elevation in all the 3 groups and on comparative evaluation, the difference in the increase in posterior and anterior elevation was significant statistically in each group (group I: p = 0.02; group II: p = 0.01; group III: p = 0.008).

The mean corneal astigmatism in group I increased from 0.89 ± 0.54D preoperatively to 2.50 ± 1.39D at 12 weeks follow-up (p= 0.004). In group II, the average corneal astigmatism increased from 0.87 ± 0.30D to 3.38 ± 2.15D at 12 weeks (p= 0.01) and in group III, the mean preoperative and postoperative corneal astigmatism was 0.85 ± 0.55D and 1.37 ± 0.87D respectively (p= 0.02). A comparative analysis of the change in corneal astigmatism following surgery between groups II & III was significant statistically (p= 0.02).

The mean preoperative axial length in group I was 23.27 ± 0.79 mm which increased to 23.98 ± 0.76 mm at 12 weeks after surgery (p= 0.009). The mean preoperative and postoperative (12 weeks follow-up) axial length in group II were 23.92 ± 1.32 mm and 25.94 ± 2.96 mm respectively (p= 0.03). The mean preoperative and postoperative axial length in group III were 22.69 ± 0.87 mm and 22.71 ± 0.83 mm respectively (p= 0.79). Comparative analysis of increase in axial length following surgery between groups I & III and groups II & III were found to be significant statistically (I & III: p = 0.003; II & III: p = 0.003).

## Discussion

Retinal surgery with or without the use of encircling and buckling elements for external tamponade can alter the shape of the globe. This may cause changes in the refractive status of the eye. Scleral buckling is known to cause a change in the shape of the sclera and can cause induced refractive changes, including astigmatic and nonastigmatic changes [5-10].

We have used Orbscan slit scanning system II to evaluate the corneal topographic changes following retinal/ vitreoretinal surgeries. The data accumulated by Orbscan may be limited by factors such as the accuracy of the system which is ± 20 μm, the measurement noise which leads to both positive and negative difference in the height of the posterior corneal surface and the necessity of aligning the posterior surface before and after surgery which may be a source of error[11,12]. However, this is the best tool available to study the posterior corneal elevation.

In the present study, there was a significant increase in both anterior and posterior corneal elevation as detected by scanning slit topography (Orbscan II topography system) following surgery. This increase in the anterior and posterior corneal elevation is probably due to the use of encircling element and/ or buckle in retinal surgeries resulting in corneal steepening. We noted that the change in the posterior elevation was more significant than anterior elevation. A comparative analysis between the three groups indicated that there was no significant difference in the anterior elevation; however the posterior elevation was significantly more in eyes with buckle. It is possible that the buckle and the encircling element have a greater effect on the posterior corneal surface. The increase in the anterior and posterior corneal elevation might be one of the contributing factors for the non improvement of visual acuity in an eye that has undergone retinal/ vitreoretinal surgery. This change might escape detection by routine videokeratography. The anterior protrusion of the corneal back surface induces an increase in the negative power of the corneal surface. Assessing the corneal surface by keratometry or placido disc videokeratography may provide inadequate information regarding refractive change caused by corneal surface alteration that results in retinal/ vitreoretinal surgery.

There was an increase in mean corneal astigmatism following surgery in all the groups. This is due to the effect of encircling element or buckle on the corneal surface. Studies have reported that induced astigmatism has been associated with radial scleral buckles[5,13], circumferential buckles[14], medial rectus disinsertion[15], anterior location of the scleral buckle[13], and use of sponge material rather than hard silicone[1]. In our study, a comparative analysis between the groups indicated that buckle causes more astigmatic changes than encircling element.

In retinal/ vitreoretinal surgeries, the encircling band creates a circular indentation of the eye, thereby increasing its anteroposterior axial length; the myopic shift may be upto 3 diopters (D)[9,10,13,16]. An increase in axial length by 0.54 mm[9] and 1.7 mm[14] has been reported following scleral buckling in two studies. In the present study, there was an increase in the mean axial length of the eyes in all the three groups (Table 1). This increase in axial length may be attributed to an anteroposterior elongation of the eyeball secondary to the transverse compression by the buckle and/ or an encircling element. The postoperative increase in axial length was found to be more pronounced in eyes with buckle. Buckle being wider and thicker results in greater indentation and thereby a greater increase in axial length than encircling element.

## Conclusions

Retinal/ vitreoretinal surgeries result in an increase in the elevation of the corneal surfaces. These changes are more pronounced on posterior corneal surface.

## Declaration of competing interest

None declared.

## Individual contribution of authors

RS designed the study and performed the data collection. NS wrote the manuscript. LKV performed the retinal surgeries. RMP performed the statistical analysis and RBV followed up the patients.

All authors read and approved the final manuscript.

## Pre-publication history

The pre-publication history for this paper can be accessed here:


